# Knockdown of Indy/CeNac2 extends Caenorhabditis elegans life span by inducing AMPK/aak-2

**DOI:** 10.18632/aging.100791

**Published:** 2015-08-20

**Authors:** Franziska Schwarz, Zehra Karadeniz, Antje Fischer-Rosinsky, Diana M. Willmes, Joachim Spranger, Andreas L. Birkenfeld

**Affiliations:** ^1^ Department of Endocrinology, Diabetes and Nutrition, Center for Cardiovascular Research, Charité – University School of Medicine, Berlin, Germany; ^2^ Section of Metabolic Vascular Medicine, Medical Clinic III and Paul Langerhans Institute Dresden (PLID), a member of the German Diabetes Center (DZD), Technische Universität Dresden, Germany; ^3^ Section of Diabetes and Nutritional Sciences, Rayne Institute, King's College London, UK

**Keywords:** Indy, CeNAC-2, aak-2, AMPK, C.elegans, Slc13A5, mIndy

## Abstract

Reducing the expression of the *Indy* (Acronym for ‘*I*'m *N*ot *D*ead, *Y*et’) gene in lower organisms promotes longevity and leads to a phenotype that resembles various aspects of caloric restriction. In *C. elegans*, the available data on life span extension is controversial. Therefore, the aim of this study was to determine the role of the *C. elegans* INDY homolog CeNAC2 in life span regulation and to delineate possible molecular mechanisms. siRNA against Indy/CeNAC2 was used to reduce expression of Indy/CeNAC2. Mean life span was assessed in four independent experiments, as well as whole body fat content and AMPK activation. Moreover, the effect of Indy/CeNAC2 knockdown in *C. elegans* with inactivating variants of AMPK (TG38) was studied. Knockdown of Indy/CeNAC2 increased life span by 22 ± 3% compared to control siRNA treated *C. elegans*, together with a decrease in whole body fat content by ~50%. Indy/CeNAC2 reduction also increased the activation of the intracellular energy sensor AMPK/aak2. In worms without functional AMPK/aak2, life span was not extended when Indy/CeNAC2 was reduced. Inhibition of glycolysis with deoxyglucose, an intervention known to increase AMPK/aak2 activity and life span, did not promote longevity when Indy/CeNAC2 was knocked down. Together, these data indicate that reducing the expression of Indy/CeNAC2 increases life span in *C. elegans,* an effect mediated at least in part by AMPK/aak2.

## INTRODUCTION

Reduced expression of the *Indy* (Acronym for ‘***I*** am ***N***ot ***D***ead, ***Y***et’) gene in *Drosophila melanogaster* promotes longevity in most studies [1–5]. The underlying mechanisms recapitulate multiple characteristics of caloric restriction, i.e. reduced in body fat, insulin like proteins [4], and increased mitochondrial biogenesis [1, 6], along with the activation of FOXO and the co-transcriptional regulator PGC-1α [1, 2, 4, 7]. Interestingly, food intake in the long lived, *Indy* mutant flies is not reduced. Knocking down the *Indy* homolog CeNAC2 in *C. elegans* also promotes a moderate increase in lifespan in one study [8], while another study failed to observe the phenotype [9]. Whether or not the knockdown of CeNAC2 really promotes longevity and by which mechanism remains unclear.

In mammals, deletion of the homolog of *Indy* (*mIndy, Slc13A5),* protects mice from high fat diet and aging induced obesity, insulin resistance and nonalcoholic fatty liver disease (NAFLD), at least in part through the activation of the intracellular energy sensor AMP-activated protein kinase A (AMPK) [6]. Life span in these mice has not yet been studied.

The *mIndy* encoded protein mINDY is part of the SLC13 protein family, consisting of Na-carboxylate and Na-sulfate cotransporters in vertebrates, invertebrates, plants, and bacteria [10, 11]. mINDY mediates the cotransport of citrate, succinate, and several other dicarboxylates across the plasma membrane together with sodium in an electrogenic manner [12]. The amino acid sequence of the N-terminal sodium and the carboxy-binding motif is highly conserved between many species, from bacteria to C. elegans to human [13]. Therefore, *C. elegans* might serve as a model entity for higher organisms.

The *C.elegans Indy* homolog CeNAC2 is expressed mostly in tissues that function as sites of nutrient absorption and fat storage [4, 12, 14]. Indy/CeNAC2 is located on the plasma membrane and transports TCA cycle intermediates, which are used to generate biochemical energy in the form of ATP. A tight regulation of net energy flux is a fundamental requirement of living organisms. Alteration of these processes can lead to profound changes in the ability of cells to cope with sudden changes in energy demand and can result in premature death. Therefore, cells possess several mechanisms to adjust and tune in energy demand and synthesis. One kinase controlling these mechanisms is AMP activated protein kinase (AMPK) [15, 16]. AMPK is activated in states of low energy supply through AMP, and it is inhibited in states of high energy abundance by ATP. Upon activation, AMPK increases mitochondrial biogenesis and shifts energy generation from glycolysis to the oxidation of fat, yielding higher ATP levels. By these and other mechanisms, the AMPK homolog aak2 mediates the life extending effect of glucose restriction in *C. elegans* [15, 17, 20].

Here, we aimed at clarifying whether or not knocking down the *C. elegans Indy* homolog CeNAC2 increases life span similar to the studies in *D. melanogaster*. We hypothesized that longevity in Indy/CeNAC2 reduced *C. elegans* is mediated via the activation of AMPK/aak2.

## RESULTS

### Effect of Indy/CeNAC2 knockdown on life span

**Figure 1:**
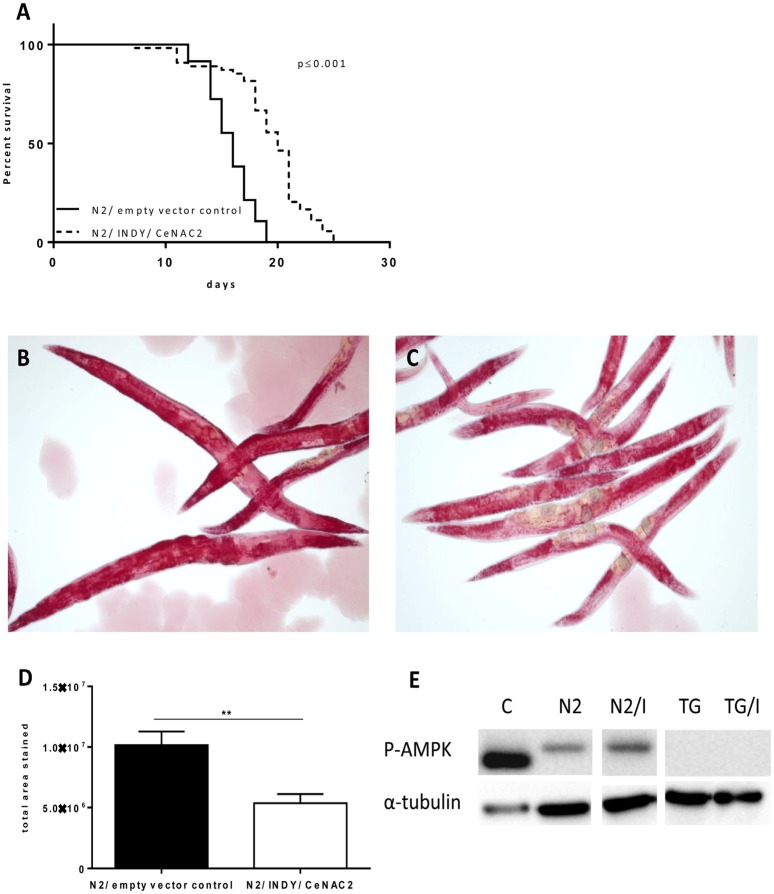
(**A**) Representative survival curves for N2 (WT), fed with *E. coli* GC363 harboring the empty vector L4400 (empty vector control) versus *E. coli* GC363 harboring INDY/CeNAC2-specific siRNA. INDY/CeNAC2-leads to a significant life span extension (*P* < 0.001). Life span experiments were repeated four times in independent experiments, figure shows the second experiment, other data in Table 1.(**B**) Oil Red O staining of N2 wild type fed with *E.coli* GC363 harboring the empty vector L4400 (empty vector control) versus *E.coli* GC363 harboring INDY/CeNAC2-specific siRNA (**C**). N2 wild type worms showed more intensive staining and higher total area stained compared to RNAi treatment (**D**), Densitometric analysis of B and C, **p = 0.005. (**E**) Immunoblot analysis of different *C. elegans* strains (N2, TG) and fasted mouse liver lysate (C) as positive control for phosphorylation (activation) of AMPK/ aak-2 after knockdown of INDY/CeNAC2 (N2/I). Down regulation of INDY/CeNAC2 leads to activation of AMPK / aak-2 compared to control fed wildtype N2. Negative control TG38 (TG) with missing phosphorylation site for AMPK-Thr172 shows no activation when fed with empty vector control neither when treated with RNAi against INDY/CeNAC2 (G/i). Control (c) = liver lysates from fasted mice.

Knockdown of Indy/CeNAC2 by feeding wildtype N2 worms with bacteria expressing Indy/CeNAC2-specific siRNA caused a significant increase in average life span of the organism (Fig. 1A). Individual data from 4 independent experiments are given in Table 1a-d. Mean life span of the worms fed on bacteria harboring Indy/CeNAC2-specific siRNA was 19.3 ± 0.52 days; mean life span of the worms fed on bacteria harboring the empty vector L4400 was 15.8 ± 0.29 days (Tab.1b). The increase in average life span induced by Indy/CeNAC2 knockdown was +22% (Log Rank *p* ≤ 0.001). A partial loss of function *daf-2* mutant strain (CB1370) was included as a positive control in the experiments and showed the documented life span extension (Table 1a-d) [18]. Thus, our studies confirm a life span extending effect of reduced Indy/CeNAC2 expression in *C.elegans*.

**Table 1:** Mean life spans in four independent experiments

Table 1a. Life-span No.1								
Strain	mean	±	SEM	n	p-value	N2 / INDY/ CeNAC2	TG38 / INDY/ CeNAC2	CB1370 / empty vector control
N2 / empty vector control	16.4	±	0.28	60		0.004		≤ 0.001
N2 / INDY/CeNAC2	17.2	±	0.36	60				≤ 0.001
TG38 / empty vector control	13.5	±	0.26	60			0.119	≤ 0.001
TG38 / INDY/CeNAC2	14.2	±	0.29	60				≤ 0.001
CB1370 / empty vector control	28.1	±	1.32	60				

Table 1b. Life-span No.2								
Strain	mean	±	SEM	n	p-value	N2 / INDY/ CeNAC2	TG38 / INDY/ CeNAC2	CB1370 / empty vector control
N2 / empty vector control	15.8	±	0.29	60		≤ 0.001		≤ 0.001
N2 / INDY/CeNAC2	19.3	±	0.52	60				≤ 0.001
TG38 / empty vector control	14.1	±	0.21	60			0.027	≤ 0.001
TG38 / INDY/CeNAC2	14.7	±	0.26	60				≤ 0.001
CB1370 / empty vector control	28.5	±	1.25	60				

Table 1c. Life-span No.3								
Strain	mean	±	SEM	n	p-value	N2 / INDY/ CeNAC2	TG38 / INDY/ CeNAC2	CB1370 / empty vector control
N2 / empty vector control	15.1	±	0.28	60		≤ 0.001		≤ 0.001
N2 / INDY/CeNAC2	17.8	±	0.39	60				≤ 0.001
TG38 / empty vector control	15.1	±	0.26	60			0.120	≤ 0.001
TG38 / INDY/CeNAC2	17.8	±	0.29	60				≤ 0.001
CB1370 / empty vector control	15.1	±	1.28	60				

Table 1d. Life-span No.4								
Strain	mean	±	SEM	n	p-value	N2 / INDY/ CeNAC2	TG38 / INDY/ CeNAC2	CB1370 / empty vector control
N2 / empty vector control	14.1	±	0.27	60		≤ 0.001		≤ 0.001
N2 / INDY/CeNAC2	15.9	±	0.27	60				≤ 0.001
TG38 / empty vector control	15.1	±	0.46	60			0.606	≤ 0.001
TG38 / INDY/CeNAC2	14.8	±	0.42	60				≤ 0.001
CB1370 / empty vector control	28.0	±	1.38	60				

Mean life span (SEM) of worms fed with *E.coli* GC363 harboring the empty vector L4400 (empty vector control) versus *E.coli* GC363 harboring INDY/CeNAC2-specific siRNA and CB1370 daf-2(e1370) fed with empty vector control. N2 wild type worms were used as control. Strain TG38 and CB1370 are short and long lived controls, respectively. n = number of worms used in the experiment. Mean life span and SEM are in days.

### Effect of Indy/CeNAC2 knockdown on body fat content

In order to test whether or not reduction of Indy/CeNAC2 in *C.elegans* also shares characteristics of caloric restriction, whole body fat stores were analyzed by phase contrast microscopy using the azo compound Oil Red O to stain triglycerides. The intensity (area by pixel) of Oil Red O staining of worms fed on bacteria harboring Indy/CeNAC2-specific siRNA was ~ 50% lower compared to control worms that were fed on bacteria harboring empty vector L4400 (Indy/CeNAC2-specific siRNA: 5.4e+006 ± 76988, control L4400: 1.016e+007 ± 1.127e+006, *p* = 0.006 Wilcoxon test) (Fig. 1B-D).

### Knockdown of CeNAC2 leads to the activation of AMPK

Given the life span extending effect of Indy/CeNAC2 knockdown together with a reduction in body fat content, we were interested in determining a unifying molecular mechanism. In mice, deletion of *mIndy* reduced the hepatocellular ATP/ADP ratio and activated hepatic AMPK [6, 19]. The *C. elegans* AMPK homolog aak2, has been shown to mediate the life span extending effect of various short-term stressors [17] including glucose restriction [20]. Phosphorylation of AMPK/aak2 at threonine 172 is widely used as a surrogate marker of activity of this kinase. Therefore, the phosphorylation of aak2 in worms fed on bacteria harboring Indy/CeNAC2 siRNA was analyzed. Western blot analysis of cell extracts was carried out using an antibody against phospho-Thr^172^ of the human AMPK. AMPK/aak2 was markedly phosphorylated in worms with reduced Indy/CeNAC2. A lower state of phosphorylation of AMPK/aak2 was observed for wildtype N2 under control conditions, fed with bacteria harboring the empty vector L4400. Blotted extracts of worms with a deletion in the aak2 gene (TG38 aak-2(gt33)), including the phosphorylation site, showed no signal for phosphorylated AMPK/aak2 (Fig. 1E).

### Effect of glucose restriction on life span in Indy/CeNAC2 knockdown

**Figure 2:**
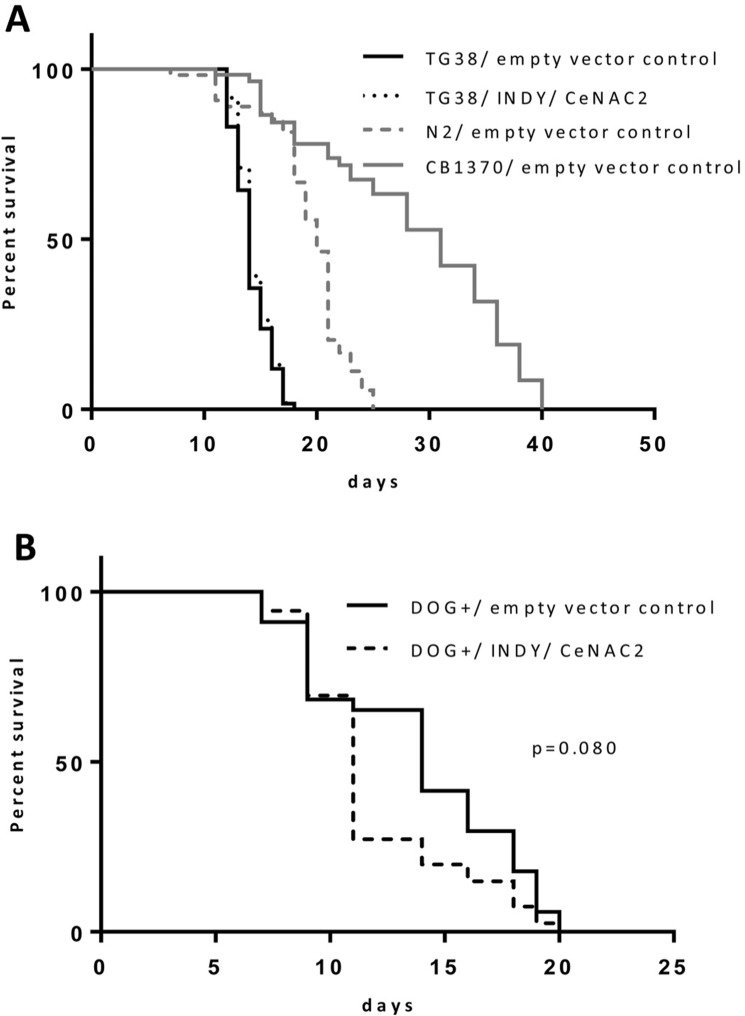
(**A**) Survival curves for TG38 AMPK/akk-2(gt33), fed with *E. coli* GC363 harboring the empty vector L4400 (empty vector control) versus *E. coli* GC363 harboring INDY/CeNAC2-specific siRNA and CB1370 daf-2(e1370) fed with empty vector control. (**B**) Survival curves for N2 (WT), fed with *E.coli* GC363 harboring the empty vector L4400 (empty vector control) versus *E.coli* GC363 harboring INDY/CeNAC2 siRNA with 2-Deoxy-D-Glucose (DOG+) in the media.

AMPK/aak2 mediates the life span extending effect of glucose restriction in C. elegans [20]. Since AMPK/aak2 was activated in Indy/CeNAC2 RNAi treated worms, and Indy/ceNAC2 encodes a transporter for citrate, a metabolite interfering with glycolysis [11, 21], altered rates of substrate flux through glycolysis might be a unifying principle to couple life span extension via AMPK to the knockdown of Indy/CeNAC2. Glycolysis can be inhibited by 2-Deoxy-D-glucose (DOG), a molecule which competitively inhibits the generation of glucose-6-phosphate from glucose at the phosphoglucokinase level. This is due to the 2-hydroxyl group replaced by hydrogen, rendering DOG incompatible for further glycolysis, while metabolism of other nutrients remains unaffected. Therefore, we tested whether or not DOG administration is still effective in increasing life span in Indy/ceNAC2 knockdown *C. elegans*. Exposure of 5 mM DOG to the nematodes caused a significant increase in average life span of the organism by 20 ± 1.3% (Log Rank *p* ≤ 0.001). Exposure of DOG to nematodes with simultaneous knockdown of the Indy homolog CeNAC2 did not show any further life span extension (Log Rank *p* = 0.135) (Fig. 2A, Tab.2a-c), suggesting that both interventions converge into well related downstream effect.

**Table 2:** Mean life spans when treated with deoxyglucose (DOG)

Table 2a. Life-span No.1							
Strain	mean	±	SEM	n	p-value	DOG− / INDY/ CeNAC2	DOG+ / INDY / CeNAC2
DOG / empty vector control	9.9	±	0.44	60		0.010	
DOG− / INDY/CeNAC2	11.8	±	0.57	60			0.841
DOG + / INDY/CeNAC2	11.6	±	0.51	60			
DOG + / empty vector control	13.4	±	0.73	60			0.080

Table 2b. Life-span No.2							
Strain	mean	±	SEM	n	p-value	DOG− / INDY/ CeNAC2	DOG+ / INDY / CeNAC2
DOG− / empty vector control	10.3	±	0.47	60		0.007	
DOG− / INDY/CeNAC2	12.2	±	0.62	60			0.432
DOG + / INDY/CeNAC2	11.7	±	0.50	60			
DOG + / empty vector control	12.9	±	0.66	60			0.217

Table 2c. Life-span No.3							
Strain	mean	±	SEM	n	p-value	DOG− / INDY/ CeNAC2	DOG+ / INDY / CeNAC2
DOG− / empty vector control	10.3	±	0.48	60		0.002	
DOG− / INDY/CeNAC2	12.7	±	0.66	60			0.119
DOG + / INDY/CeNAC2	11.6	±	0.47	60			
DOG + / empty vector control	12.9	±	0.69	60			0.109

Mean life span of worms fed with *E. coli* GC363 harboring the empty vector L4400 (empty vector control) versus *E. coli* GC363 harboring INDY/CeNAC2-specific siRNA when treated with DOG. Mean life span and SEM are in days.

### Role of AMPK/aak2 in the life span extending effect of Indy/CeNAC2

In order to determine whether or not the life span extending effect of the reduction of Indy/CeNAC2 depends on AMPK/aak2, life span studies were repeated in *C. elegans* with an inactivating mutation of AMPK/aak2, TG38 aak-2(gt33). In these TG38 aak-2(gt33) worms, RNAi-mediated knock down of Indy/CeNAC2 did not increase average life span, while we continued to observe the life span extending effect in the N2 wild type (Fig. 2A and Tab.1a-d). Mean life span of TG38 aak-2(gt33)/empty vector control did not differ from TG38 aak-2(gt33)/ Indy/CeNAC2 in all experiments. Long-lived CB1370 daf-2 (e1370) worms, a positive control for life span extension, showed the expected increase in life span in all experiments (Tab.1a-d). To confirm that the mutation in AMPK/aak2 did not by itself alter the expression of Indy/CeNAC2, expression levels were determined by qPCR. Expression levels of Indy/CeNAC2 in TG38 (aak-2(gt33)) Indy/CeNAC2 siRNA treated worms were lowered by 58% (*p* = 0.05) compared to TG38 aak-2(gt33)/empty vector control treated worms.

## DISCUSSION

Our studies show that knockdown of Indy/CeNAC2 in C. elegans leads to a significant increase in life span. AMPK/aak2 was activated in CeNAC2 reduced *C. elegans*. Moreover, in worms with inactivated AMPK, knockdown of CeNAC2 was not able to extend life span. Thus, our data suggest that the life span extending effect of knockdown of Indy/CeNAC2 is mediated via AMPK, at least in part.

A previous study did not observe a life span extending effect of Indy/CeNAC2 knockdown in *C. elegans* nor a life span increase with *Indy* knockdown in D. melanogaster [9]. Subsequent studies in *D. melanogaster* have resolved this discrepancy in flies by demonstrating that knockdown of *Indy* in flies is mediated by the induction of a calorie restriction-like state [4], as it is in mice [6]. A possible explanation might be that the two studies used different diets, with the study that did not observe a life span extension used a diet in flies that resembled more a low calorie diet [4, 9, 22]. In this scenario, it is possible that the knockdown of Indy does not extend life span any further.

In the study presented here, *E.coli* GC363 harboring the empty vector L4400 or the vector with the CeNAC2-specific siRNA was used. Using this regular, commonly used food [23], we observed a life span extending effect with the knock down of Indy/CeNAC2 in *C. elegans* for mean, median and maximum life span (see supplemental data Table S3 and S5). Together with life span extension, Indy/CeNAC2 Knockdown also resulted in reduced whole body fat stores in our worms. This finding is in line with observations in *D. melanogaster* [4], *C. elegans* [8] and mice [6]. The reduction in body fat might be mediated via multiple mechanisms. First, the functional role of Indy/CeNAC2 is to import citrate as well as several dicarboxylates into the cytosol as substrates for several metabolic processes [8]. For example, citrate is the main carbon source of fatty acid and cholesterol synthesis, and it acts as an allosteric activator of acetyl-CoA carboxylase (ACC) [11]. Moreover, cytosolic citrate furnishes NADPH generation via malic enzyme for lipogenesis [24]. Overexpression of mammalian Indy homolog in a HepG2 cell line resulted in an increase in intracellular fatty acid and sterol synthesis in our previous studies [25]. In line with this notion, enhancing the activity of citrate transport by mINDY in HepG2 cells has been shown to enhance citrate induced lipid synthesis [25, 26], while knocking down mINDY in hepatocytes leads to a reduction in hepatocyte lipid content [6, 27]. It is possible that reducing the import of citrate by knocking down Indy/CeNAC2 reduces lipid synthesis directly by reducing citrate as a C-donor for fatty acid synthesis as well as an allosteric activator for lipid synthesis [27].

Moreover, we observed that reducing Indy/CeNAC2 expression is associated with the activation of the intracellular energy sensor AMPK/aak2. When reducing Indy/CeNAC2 expression in AMPK/aak2 mutated *C. elegans* TG38 aak-2(gt33), which do not possess the ability to phosphorylate, and thus, activate AMPK, the life span extending effect of the Indy/CeNAC2 KD was not observed any longer.

AMPK has previously been shown to mediate longevity in *C. elegans* when glucose is restricted as a nutritional substrate [20]. In this case, AMPK is activated via AMP. Moreover, AMPK has also been put into context to mediate the longevity effect of SIRT1 and metformin in *C. elegans* and mice [15,16,28]. Moreover, metformin might activate AMPK via inhibition of complex 1 of the respiratory chain, reducing ATP generation and increasing the AMP/ATP ratio. Other mechanisms contribute [29]. The inability to increase lipid oxidation in aging rats has been attributed to reduced activation of AMPK [30]. Finally, in mice with deleted mINDY, the ATP/AMP ratio is shifted towards AMP, and ATP levels are largely reduced [6, 31]. Consecutively, AMPK is activated when INDY is knocked out in mice. Collectively, these data provide a rationale for the molecular mechanisms mediating longevity when Indy/CeNAC is reduced.

In our studies, reducing glucose availability through DOG did not enhance life span any further in Indy/CeNAC2 knock down, as shown for mean, median and maximum life span (see supplemental data Table S4 and S6). These data further strengthen the role of AMPK in mediating the life span extending effect of Indy/CeNAC2 reduction, since inhibition of glycolysis using DOG in *C. elegans* has been shown to lead to an extended life span by activating AMPK [20]. Findings from Neretti and colleagues suggested that when INDY is reduced, a decreased flux of electrons through the electron transport chain associated with an upregulation of mitochondrial biogenesis combine to result in an overall decrease of reactive oxygen species and maintenance of normal cellular ATP levels [1]. Clearly, a thorough analysis of the metabolic pathways that are altered by the deletion and/or reduction of Indy/CeNAC2 are needed to better understand how AMPK/aak2 might be activated.

In summary, knockdown of Indy/CeNAC2 in *C. elegans* extends life span along with the activation of the intracellular energy sensor AMPK. Our data support the hypothesis that AMPK is indispensable for Indy/CeNAC2 to mediate longevity and provide a rationale for the molecular mechanisms mediating longevity when Indy/CeNAC2 is reduced. Together, these data suggest that reducing the expression or activity of the mammalian homolog of the tri- and dicarboxylic acid transporter Indy/CeNAC2 might be an attractive target to promote healthy aging by activating AMPK and, possibly, to extend life span.

## METHODS

### Strains and culture conditions

*C. elegans* strains Wild-type N2, TG38 *aak-2(gt33)* and *E.coli* OP50 and GC363 were obtained from the *Caenorhabditis* Genetics Center (Minneapolis, MN). The *gt33* allele has a 606-bp deletion. Wild-type N2 and *aak-2* mutant worms were maintained at 22°C on Nematode Growth Medium (NGM) plates seeded with *E. coli* OP50 using standard methods [32] except for experiments where other is specified.

### Bacterially-mediated RNAi

Genetically modified bacteria strain (*E. coli* HT115 ) for CeINDY was retrieved from Source BioScience. Carrying the L4400 vector, which contains convergent T7 polymerase promoters separated by a multicloning site. *E. coli* GC363 carry the empty L4400 vector and are considered as the control. Control and siRNA expressing *E.coli* strains were grown over night on LB Agar at 37°C containing 100 μg/ml ampicillin and 12.5 μg tetracyclin. A single colony was picket and transferred into LB-Media and cultivated over night for a starter culture. Preceding every experiment for bacterial-mediated RNAi, a fresh culture was grown for 6 h in LB-Media from an aliquot of the starter culture. 4–7 days ahead of every experiment the according NGM plates were prepared containing 100 μg/ml ampicillin (Roth) and 1 mM Isopropyl-β-D-thiogalactopyranoside (IPTG, Sigma) as well as 0.1 mg/ml 5-fluoro-2′-deoxyuridine (FUDR, Sigma) if needed.

### Life span assays

Worms were cultured at 20°C on Nematode Growth Medium (NGM) plates seeded with *E. coli* OP50 using standard methods. Prior to the experiments worms were synchronized via 20% Alkaline Hypochlorite Solution using standard protocol [32], eggs were transferred to new plates seeded with OP50. At the L4 (*t* = 0) moult, animals were transferred to plates containing ampicillin, FUDR and IPTG. RNAi expressing bacteria, were received as sequence verified strains by BioSource (Nottingham, GB). RNAi mediated knockdown of CeNAC2 in N2 was conducted as described by Fei et al. 2003 with significant down regulation of gene expression. As mentioned above seeded with RNAi expressing bacteria or control bacteria (empty vector control). Animals were transferred to new plates every second day and counted. Animals were judged dead when they did not move after repeated prodding with a pick. Animals that crawled off the plates were not included in the analysis (Kaplan Meyer). Assays were repeated independently four times for validation. Plates for life span assay with DOG were prepared from the same bath of NGM agar. The respective chemical was added to a final concentration of 5 mM from a sterile 0.5 M stock solution for DOG. DOG treatment lasted for seven days after larvae reached the L4 moult. Assay was conducted until all worms were either censored or counted as dead as described above.

### Expression

Parallel to life span assays nematodes were cultured on plates containing ampicillin, FUDR and IPTG as mentioned above for 5 days, regularly fed with siRNA expressing bacteria. After 5 days worms were washed off plates with M9-Buffer and shortly centrifuged. Supernatant was removed and 500 μl TRIZOL (Life Technologies) was added to the worm pellet. For lysis of worms a Speed Mill (Analytik Jena) was used. Samples were centrifuged for 5 min at full speed, supernatant was transferred into a new tube and 100 μl was added per 500 μl of supernatant volume. Followed by an incubation for 2 min on ice, the samples were centrifuged for 15min at 4°C (12000xg). The polar phase was removed and the nucleic acids were precipitated via isopropyl and washed with ethanol (70%). RNA was resuspended in nuclease free water. Residues of DNA were digested using DNase I, Rnase-free (Thermo Scientific), for cDNA synthesis RevertAid First Strand cDNA Synthesis Kit was used. For detection SYBR Green and 7300 Real Time RCE System by Apllied Biosystems was used. Once knockdown was established it was checked periodically.

### Western blot analysis

Synchronized Wild-type N2 and *aak-2(gt33)* eggs were transferred to NGM plates with GC363 as the main food source. At 22°C eggs were allowed to hatch und larvae to grow until L4 stage. At the first adult day worms were transferred to NGM-plates containing IPTG seeded with siRNAi producing bacteria. After 4 days of feeding, worms were washed from NGM plate using M9 buffer. Worms were set on ice to assemble on the bottom of the tube. After removing the supernatant, the worms were transferred to a tube containing beeds (Carl Roth GmbH) for lysis (Speed Mill Plus, Analytik Jena) and 2 volumes RIPA buffer (150 mM NaCl, 10 nM Tris, pH 7.2, 1% Triton X-100, 1% Desoxycholate, 5 mM EDTA) was added to the pellet. After lysis samples were centrifuged at maximum speed for 5 min (4°C) and supernatant was transferred into a fresh tube. Protein concentration was determined via BCA-Assay (Thermo Scientific). 10 μg of protein from starved mouse liver lysate (C) as positive control was used along with 30 μg of protein from each lysed worm sample for electrophoresis. Samples were electrophoresed on an 10% SDS-polyacrylamide gel and electroblotted onto a PVDF membrane (Milipore). The membrane was incubated in a blocking solution containing anti-phospho-AMPK/ anti-AMPK antibody (1:1000dilution; #2535/ #2532, Cell Signaling Technology) or anti-tubulin mouse antibody (1:1000 dilution; #ab7291, Abcam) at 4°C overnight, followed by incubation with anti-mouse or anti-rabbit horseradish peroxidase antibody (1:5000 dilution; #7076/ #7074S Cell Signaling Technology) for 2 h at 25°C. Stripping Reagent by Roche was used. The ECL Western blotting analysis system (Roche) was used to detect the secondary antibodies on the membrane. Luminescence of the blot was captured using Chemidoc imaging system (Bioad).

### Oil red O staining

Worms were cultured at 20°C on Nematode Growth Medium (NGM) using standard methods. Prior to the experiments worms were synchronized via 20% Alkaline Hypochlorite Solution using standard protocol [32], eggs were transferred to new plates for hatching as previously described for life span assays. At the L4 (*t* = 0) moult, animals were transferred to plates containing ampicillin, FUDR and IPTG and stained with Oil Red O after 7 days. For Oil Red O staining, animals were washed in M9 buffer, and fixed with 4% paraformaldehyde for 15 minutes, were washed again and stained in filtered Oil Red O staining solution (60% Oil Red O stock solution (5 mg/ml isopropanol)/ 40% distilled water) for 30 minutes. After washing in M9, worms were analyzed using BZ-II Analyzer (KEYENCE, Germany) microscope. Photographs of 8–12 worms per condition were taken and at 40x magnification at a fixed exposure time.

### Statistical analysis

Statistical analyses for Indy/CeNAC2 knock down and fat content were performed by Wilcoxon test. For comparing significant distributions between different groups in the life span assays statistical calculations were carried out using the log-rank rest (Kaplan Meier) as well as the χ^2^-Test. Given data shows mean values ±SEM if not described otherwise. All calculations were performed using Microsoft Office Excel 2010 and SPSS version 20.0. Experiments were performed in triplicate except where stated otherwise. Error measures were calculated form the variability of the independently prepared biological samples of the same treatment group where applicable. Differences were considered statistically significant at *p* < 0.05, therey indicating a probability of error lower than 5%.

## SUPPLEMENTAL DATA TABLES


