# Supplementary material for: Knockdown of Indy/CeNac2 extends Caenorhabditis elegans life span by inducing AMPK/aak-2
Source: Aging (Albany NY). 2015 Aug 20;7(8):553–62. doi: 10.18632/aging.100791 (PMC4586101; doi:10.18632/aging.100791)
Supplement: Supplementary file 1 [file aging-07-553-s001.pdf]

SUPPLEMENTAL DATA

Table S1. Mean life spans in four independent experiments.

Table S1e. Life-span No.1

Strain	mean	±	SEM	n	p-value χ^2	N2 / INDY/ CeNAC2	TG38 / INDY/ CeNAC2	CB1370 / empty vector control
N2 / empty vector control	16.4	±	0.28	60		0.027		≤0.001
N2 / INDY/CeNAC2	17.2	±	0.36	60				≤0.001
TG38 / empty vector control	13.5	±	0.26	60			≤0.001	≤0.001
TG38 / INDY/CeNAC2	14.2	±	0.29	60				≤0.001
CB1370 / empty vector control	28.1	±	1.32	60				

Table S1f. Life-span No.2

Strain	mean	±	SEM	n	p-value χ^2	N2 / INDY/ CeNAC2	TG38 / INDY/ CeNAC2	CB1370 / empty vector control
N2 / empty vector control	15.8	±	0.29	60		≤0.001		≤0.001
N2 / INDY/CeNAC2	19.3	±	0.52	60				≤0.001
TG38 / empty vector control	14.1	±	0.21	60			≤0.001	≤0.001
TG38 / INDY/CeNAC2	14.7	±	0.26	60				≤0.001
CB1370 / empty vector control	28.5	±	1.25	60				

Table S1g. Life-span No.3

Strain	mean	±	SEM	n	p-value χ^2	N2 / INDY/ CeNAC2	TG38 / INDY/ CeNAC2	CB1370 / empty vector control
N2 / empty vector control	15.1	±	0.28	60		≤0.001		≤0.001
N2 / INDY/CeNAC2	17.8	±	0.39	60				≤0.001
TG38 / empty vector control	15.1	±	0.26	60			0.131	≤0.001
TG38 / INDY/CeNAC2	17.8	±	0.29	60				≤0.001
CB1370 / empty vector control	15.1	±	0.28	60				

Table S1h. Life-span No.4

Strain	mean	±	SEM	n	p-value χ^2	N2 / INDY/ CeNAC2	TG38 / INDY/ CeNAC2	CB1370 / empty vector control
N2 / empty vector control	14.1	±	0.27	60		0.001		≤0.001
N2 / INDY/CeNAC2	15.9	±	0.27	60				≤0.001
TG38 / empty vector control	15.1	±	0.46	60			0.597	≤0.001
TG38 / INDY/CeNAC2	14.8	±	0.42	60				≤0.001
CB1370 / empty vector control	28.0	±	1.38	60				

Mean life span (±SEM) of worms fed with *E. coli* GC363 harboring the empty vector L4400 (empty vector control) versus *E.coli* GC363 harboring the INDY/CeNAC2 RNAi vector and CB1370 daf-2(e1370) fed with empty vector control. N2 wild type worms were used as control. Strain TG38 is short lived. Strain CB1370 served as long lived control. n = number of worms used in the experiment. Mean life span and SEM are in days. Experiments were performed four times with 15 worms per group (total 60). χ^2-Test was performed using IMB SPSS Statistics 20.

Table S2. Mean life spans when treated with deoxyglucose (DOG).

Table S2d. Life-span No.1

Strain	mean	±	SEM	n	p-value χ^2	DOG- / INDY/ CeNAC2	DOG+ / INDY / CeNAC2
DOG - / empty vector control	9.9	±	0.44	60		0.084	
DOG - / INDY/CeNAC2	11.8	±	0.57	60			0.401
DOG + / INDY/CeNAC2	11.6	±	0.51	60			
DOG + / empty vector control	13.4	±	0.73	60			0.211

Table S2e. Life-span No.2

Strain	mean	±	SEM	n	p-value χ^2	DOG- / INDY/ CeNAC2	DOG+ / INDY / CeNAC2
DOG - / empty vector control	10.3	±	0.47	60		0.182	
DOG - / INDY/CeNAC2	12.2	±	0.62	60			0.882
DOG + / INDY/CeNAC2	11.7	±	0.50	60			
DOG + / empty vector control	12.9	±	0.66	60			0.297

Table S2f. Life-span No.3

Strain	mean	±	SEM	n	p-value χ^2	DOG- / INDY/ CeNAC2	DOG+ / INDY / CeNAC2
DOG - / empty vector control	10.3	±	0.48	60		0.273	
DOG - / INDY/CeNAC2	12.7	±	0.66	60			0.614
DOG + / INDY/CeNAC2	11.6	±	0.47	60			
DOG + / empty vector control	12.9	±	0.69	60			0.027

Life span represents the mean of four independent experiments with 15 worms per group (total 60). Mean life span of worms in days ± SEM fed with *E.coli* GC363 harboring the empty vector L4400 (empty vector control) versus *E.coli* GC363 harboring INDY/CeNAC2- siRNA when treated with DOG. Mean life span and SEM are in days.

Table S3. Mean, median and maximum life span data

Table S3a. Life-span No.1

strain	mean	±	SEM	n	median	±	SEM	maximum life span	±	SEM
N2 / empty vector control	16,4	±	0,28	60	17,0	±	0,36	19,0	±	.
N2 / ceINDY	17,2	±	0,36	60	17,0	±	0,58	21,0	±	.
TG38 / empty vector control	13,5	±	0,26	60	14,0	±	0,39	16,5	±	0,67
TG38 / ceINDY	14,2	±	0,29	60	14,0	±	0,38	17,5	±	0,22
CB1370 / empty vector control	28,1	±	1,32	60	31,0	±	2,06	39,5	±	0,50

Table S3b. Life-span No.2

strain	mean	±	SEM	n	median	±	SEM	maximum life span	±	SEM
N2 / empty vector control	15,8	±	0,29	60	16,0	±	0,42	18,8	±	0,17
N2 / ceINDY	19,3	±	0,52	60	20,0	±	0,39	24,5	±	0,22
TG38 / empty vector control	14,1	±	0,21	60	14,0	±	0,24	17,0	±	.
TG38 / ceINDY	14,7	±	0,26	60	15,0	±	0,42	17,7	±	0,21
CB1370 / empty vector control	28,5	±	1,25	60	31,0	±	2,03	39,3	±	0,42

Table S3c. Life-span No.3

strain	mean	±	SEM	n	median	±	SEM	maximum life span	±	SEM
N2 / empty vector control	15,1	±	0,28	60	15,0	±	0,51	18,3	±	0,33
N2 / ceINDY	17,8	±	0,39	60	19,0	±	0,49	21,3	±	0,33
TG38 / empty vector control	15,1	±	0,26	60	14,0	±	0,39	17,5	±	0,34
TG38 / ceINDY	17,8	±	0,29	60	14,0	±	0,38	17,3	±	0,33
CB1370 / empty vector control	28,0	±	1,32	60	31,0	±	2,14	39,7	±	0,33

Table S3d. Life-span No.4

strain	mean	±	SEM	n	median	±	SEM	maximum life span	±	SEM
N2 / empty vector control	14,1	±	0,27	60	14,0	±	0,24	17,7	±	0,42
N2 / ceINDY	15,9	±	0,27	60	16,0	±	0,48	18,8	±	0,31
TG38 / empty vector control	15,1	±	0,46	60	16,0	±	0,79	19,3	±	0,21
TG38 / ceINDY	14,8	±	0,42	60	15,0	±	0,70	19,3	±	0,33
CB1370 / empty vector control	28,0	±	1,38	60	30,0	±	2,54	39,7	±	0,56

Mean, median and maximum life span in days ± SEM of nematodes fed with *E.coli* GC363 harboring the empty vector L4400 (empty vector control) versus *E.coli* GC363 harboring INDY/CeNAC2 siRNA and CB1370 daf-2(e1370) fed with empty vector control. N2 wild type worms were used as control. Strain TG38 is short lived. Strain CB1370 served as long lived control. Experiments were perforned four times with 15 worms per group (total 60).

Table S4. Mean, median and maximum life span data

Table S4a. Life-span No.1

strain	mean	±	SEM	n	median	±	SEM	maximum life span	±	SEM
DOG - / empty vector control	9,9	±	0,44	60	9,0	±	0,26	15,7	±	0,61
DOG - / ceINDY	11,8	±	0,57	60	11,0	±	0,55	18,0	±	0,73
DOG + / ceINDY	11,6	±	0,51	60	11,0	±	0,32	18,0	±	0,45
DOG + / empty vector control	13,4	±	0,73	60	14,0	±	1,29	17,7	±	0,56

Table S4b. Life-span No.2

strain	mean	±	SEM	n	median	±	SEM	maximum life span	±	SEM
DOG - / empty vector control	10,3	±	0,47	60	9,0	±	0,29	15,7	±	0,61
DOG - / ceINDY	12,2	±	0,62	60	11,0	±	0,51	19,0	±	0,37
DOG + / ceINDY	11,7	±	0,50	60	11,0	±	0,36	17,8	±	0,48
DOG + / empty vector control	12,9	±	0,66	60	14,0	±	1,42	17,2	±	0,48

Table S4c. Life-span No.3

strain	mean	±	SEM	n	median	±	SEM	maximum life span	±	SEM
DOG - / empty vector control	10,3	±	0,48	60	9,0	±	0,31	15,7	±	0,61
DOG - / ceINDY	12,7	±	0,66	60	11,0	±	0,63	18,7	±	0,21
DOG + / ceINDY	11,6	±	0,47	60	11,0	±	0,36	17,3	±	0,33
DOG + / empty vector control	12,9	±	0,69	60	14,0	±	1,43	17,5	±	0,56

Mean, median and maximum life span in days ± SEM of nematodes fed with *E. coli* GC363 harboring the empty vector L4400 (empty vector control) versus *E. coli* GC363 harboring INDY/CeNAC2- siRNA when treated with DOG. Experiments were performed 4 times with 15 worms per group (total 60).

Table S5. Percent median and maximum life span compared to controls

	% mean	±	SEM	% median	±	SEM	% max	±	SEM
**N2 / empty vector control**	100			100			100		
**N2 / ceINDY**	14,34	±	3,65	16,49	±	6,14	15,90	±	5,14
**TG38 / empty vector control**	100			100			100		
**TG38 / ceINDY**	8,32	±	3,16	3,57	±	2,06	2,26	±	1,15

Percent median and maximum life span compared to controls (±SEM) under DOG treatment, n=60. Maximum life span is the mean life span of the most long-lived 10%; n=6

Table S6 Percent median and maximum life span compared to controls

	% mean	±	SEM	% median	±	SEM	% max	±	SEM
**DOG - / empty vector control**	100			100			100		
**DOG - / ceINDY**	20,12	±	1,28	22,22	±	0,00	18,44	±	1,63
**DOG + / empty vector control**	100			100			100		
**DOG + / ceINDY**	-10,99	±	1,06	-21,43	±	0,00	1,61	±	1,22

Percent median and maximum life span compared to controls (±SEM) under DOG treatment, n=60. Maximum life span is the mean life span of the most long-lived 10%; n=6
